# Breast Cancer Case Identification Based on Deep Learning and Bioinformatics Analysis

**DOI:** 10.3389/fgene.2021.628136

**Published:** 2021-05-17

**Authors:** Dongfang Jia, Cheng Chen, Chen Chen, Fangfang Chen, Ningrui Zhang, Ziwei Yan, Xiaoyi Lv

**Affiliations:** ^1^College of Information Science and Engineering, Xinjiang University, Urumqi, China; ^2^Key Laboratory of Signal Detection and Processing, Xinjiang University, Urumqi, China

**Keywords:** breast cancer, SVM, ANN, WGCNA, PPI

## Abstract

Mastering the molecular mechanism of breast cancer (BC) can provide an in-depth understanding of BC pathology. This study explored existing technologies for diagnosing BC, such as mammography, ultrasound, magnetic resonance imaging (MRI), computed tomography (CT), and positron emission tomography (PET) and summarized the disadvantages of the existing cancer diagnosis. The purpose of this article is to use gene expression profiles of The Cancer Genome Atlas (TCGA) and Gene Expression Omnibus (GEO) to classify BC samples and normal samples. The method proposed in this article triumphs over some of the shortcomings of traditional diagnostic methods and can conduct BC diagnosis more rapidly with high sensitivity and have no radiation. This study first selected the genes most relevant to cancer through weighted gene co-expression network analysis (WGCNA) and differential expression analysis (DEA). Then it used the protein–protein interaction (PPI) network to screen 23 hub genes. Finally, it used the support vector machine (SVM), decision tree (DT), Bayesian network (BN), artificial neural network (ANN), convolutional neural network CNN-LeNet and CNN-AlexNet to process the expression levels of 23 hub genes. For gene expression profiles, the ANN model has the best performance in the classification of cancer samples. The ten-time average accuracy is 97.36% (±0.34%), the F1 value is 0.8535 (±0.0260), the sensitivity is 98.32% (±0.32%), the specificity is 89.59% (±3.53%) and the AUC is 0.99. In summary, this method effectively classifies cancer samples and normal samples and provides reasonable new ideas for the early diagnosis of cancer in the future.

## Introduction

Currently, breast cancer (BC) becomes one of the most common cancers among American women, accounting for approximately one-third of all cancers. BC is the second leading cause of female cancer deaths after lung cancer (DeSantis et al., 2014). According to a report released by the International Agency for Research on Cancer in 2018, there were 9.6 million cancer-related deaths in 2018, of which 11.6% were BC in women (Bray et al., 2018). There are many deaths from BC, and its incidence is higher, especially in developed countries (Key et al., 2001). The most important environmental factors that lead to a high incidence are exposure to ionizing radiation and combined postmenopausal hormone therapy (Smith-Bindman, 2012). If people, unfortunately, have BC, doctors will use different treatment methods under the different stages of the disease (Miller et al., 2019). In short, the main methods include: radiotherapy (Balaji et al., 2016), surgery (De La Cruz et al., 2016), and chemotherapy (Ithimakin and Chuthapisith, 2013; Karagiannis et al., 2017).

The early diagnosis of cancer can improve the effectiveness of treatment. Currently, imaging diagnosis of cancer includes Mammography, Ultrasound, magnetic resonance imaging (MRI), computed tomography (CT), and positron emission tomography (PET). Among them, mammography, CT, and PET have the risk of radiation; Mammography, Ultrasound, and CT have low sensitivity (Wang, 2017). Pathological diagnosis of cancer is not suitable for rapid diagnosis due to the shortage of doctors and the large workload of manual diagnosis (Cui et al., 2019). The cancer sample classification method based on gene expression profile can conduct BC diagnosis more rapidly with high sensitivity and have no radiation (Zhang et al., 2020).

With the rapid development of bioinformatics, we can solve problems at the molecular level (Can, 2014). Gene modules related to clinical features can be screened out by WGCNA, which plays a key role in discovering genes related to human cancer (Saris et al., 2009; Yang et al., 2014; Li et al., 2018). At present, WGCNA has been applied to the analysis of various cancers, e.g., bladder cancer (Di et al., 2019), BC (Jia et al., 2020), and lung cancer (Niemira et al., 2019). Gene differential expression analysis (DEA) is another method of analyzing marker genes and has been applied to detect marker genes of various cancers, e.g., colorectal cancer (Hamfjord et al., 2012). The gene DEA software packages include Cuffdiff (Trapnell et al., 2013), edgeR (Robinson et al., 2010), and limma (Smyth, 2004). The appropriate software package can be chosen according to the research needs (Rapaport et al., 2013). Currently, WGCNA and DEA can be used together to screen out gene clusters related to the research target (Huang et al., 2019). PPI network can be used to analyze the interaction relationship between proteins. Simultaneously, it can be used to screen out hub genes related to cancer tissue proteins (Liu et al., 2009). The expression level of hub genes can be analyzed by deep learning (Khan et al., 2001; Rahman and Adjeroh, 2019; Zeng et al., 2019; Mallik et al., 2020). This analysis can achieve good results at the genetic level. Therefore, we can use it to classify cancer samples and normal samples. This study is also of great significance to the diagnosis of cancer in the future.

## Materials and Methods

### Materials

Breast cancer gene expression profiles were downloaded from The Cancer Genome Atlas (TCGA)^1^ and Gene Expression Omnibus (GEO)^2^ databases.

When the BC data set was downloaded based on the HTSeq-counts workflow through the TCGA database, 1,222 samples were obtained, including 1,109 cancer patients and 113 normal controls. Besides, another batch of gene expression profile data was from the GEO database, and its gene chip was GSE15852 including 43 normal and 43 cancer samples.

With reference to the selection of DEM, we designed a way to screen gene expression (Mallick et al., 2020). Primarily, we extracted the corresponding gene expressions according to the gene ID from the original data. Then, we replaced the missing gene expression with 0 and merged the same data. According to the count-per-million (cpm < 1), some invalid values and the impact of sequencing depth were excluded. In the end, 14,902 gene expressions of each sample were selected from TCGA, and 12,548 genes of each sample were selected from GEO.

### Methods

This study first selected the genes most relevant to cancer through weighted gene co-expression network analysis (WGCNA) and DEA. Then it used the protein–protein interaction (PPI) network to screen 23 hub genes. Finally, it used the support vector machine (SVM), decision tree (DT), Bayesian network (BN), artificial neural network (ANN), convolutional neural network CNN-LeNet and CNN-AlexNet to process the expression levels of 23 hub genes.

**FIGURE 1:**
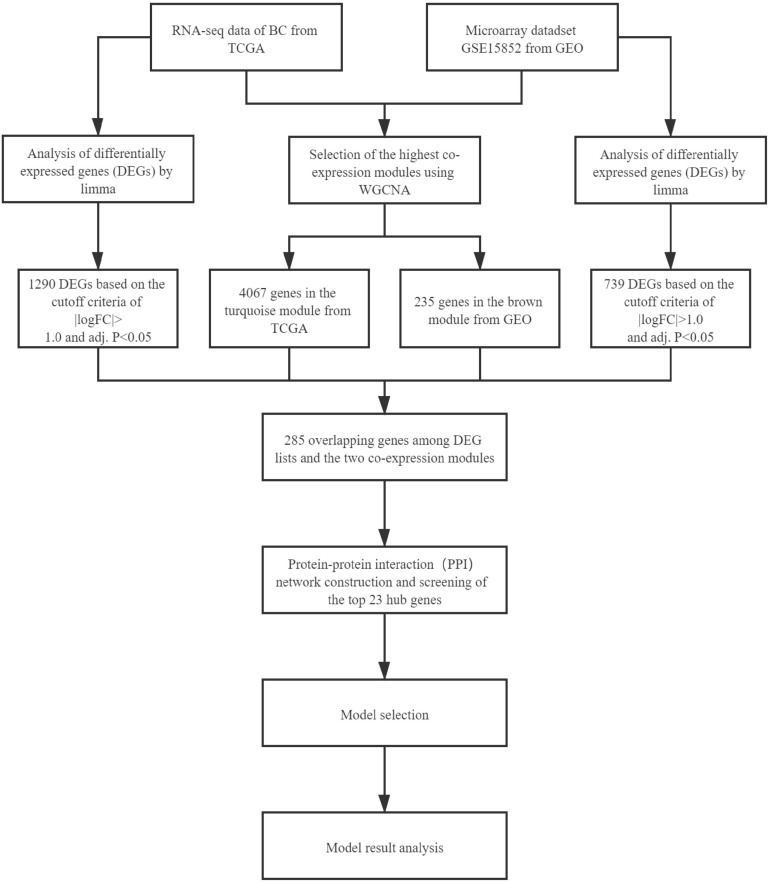
Workflow of this study.

The workflow of this study is shown in Figure 1. We describe the methods used in the figure as following.

The gene modules were screened by WGCNA. After the gene expression profile was obtained, the WGCNA software package in R (Langfelder and Horvath, 2008) was used to configure the gene expression data of GSE15852 and TCGA-BC as a gene co-expression network. The adjacency matrix of WGCNA is *A*_*ij*_ = |*S*_*ij*_|^β^ (*A*_*ij*_ is the adjacency matrix between gene *i* and gene *j*, *S*_*ij*_ is the Pearson coefficient of similarity matrix of all gene pairs, and β is the soft power value). *A*_*ij*_ was converted into corresponding dissimilarity of topological overlap matrix (CD-TOM). Gene modules were classified by CD-TOM hierarchical clustering. To explore the relationship between gene modules and clinical features, this study calculated the correlation coefficients between modules and clinical features. The gene module with highest correlation coefficient was selected for the subsequent analysis.

The differentially expressed genes (DEGs) were screened by limma. The limma in the R software package provides a solution for the DEA of microarray data. This study used limma to screen DEGs between normal tissues and BC tissues in the GSE15852 and TCGA-BC datasets, respectively. The *P-*value was adjusted by the Benjamini-Hochberg method to control the false discovery rate (FDR). Both |logFC| ≥ 1.0 and adjusted *P* < 0.05 were used as the thresholds for DEGs. All DEGs were visualized by a volcano plot.

The PPI network selected hub genes from overlapping genes and it was built by the STRING database. Public genes in DEGs and co-expressed genes were used as overlapping genes, and overlapping genes were visualized by the R package Venn diagram. The overlapping genes were used for PPI network construction, and hub genes were extracted according to the maximal clique centrality (MCC) rule.

As classification model selection, in this study, SVM, BN, and DT models were selected in machine learning, and ANN, CNN-LeNet, and CNN-AlexNet were selected in deep learning. The expression level of 23 hub genes of the sample was the entire data set. The dataset was randomly classified into the training set (70%) and the test set (30%). All algorithms were trained on the training set, and the classification results were obtained from the test set. With the average accuracy as the initial standard, we selected the two models with higher accuracy. To get the best classification model, a comparative analysis was performed in the two models. In the end, we obtained the optimal model for cancer diagnosis.

## Results

**FIGURE 2:**
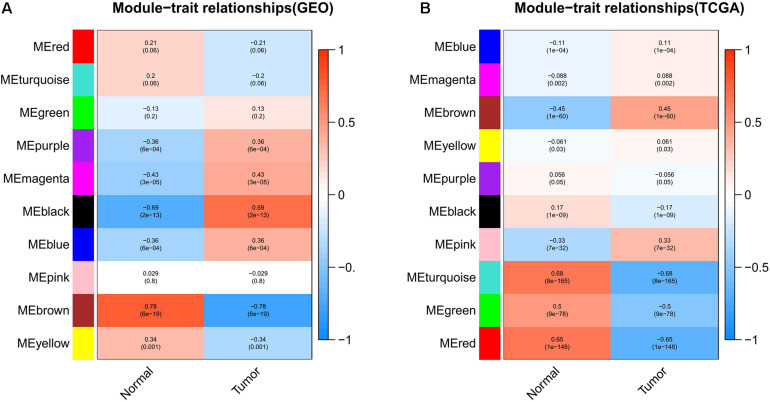
Module-trait relationships in the **(A)** GEO and **(B)** TCGA. Each row represents a gene module. Each column corresponds to the clinical characteristics of the cancer. Each grid contains the correlation coefficient and *P*-value of the gene module.

Weighted gene co-expression network analysis can be used to screen out the gene modules related to cancer tissues. The module-trait relationships of the GSE15852 and TCGA-BC datasets are shown in Figures 2A,B, respectively. The genes were divided into 10 parts. The genes of each part had been matched with different colors. To select the gene module that best matches the clinical characteristic, we chose the module with the highest correlation coefficient. The MEbrown module, which contained 235 genes, was selected in the GEO. The MEturquoise module, which contained 4,067 genes, was selected in the TCGA.

**FIGURE 3:**
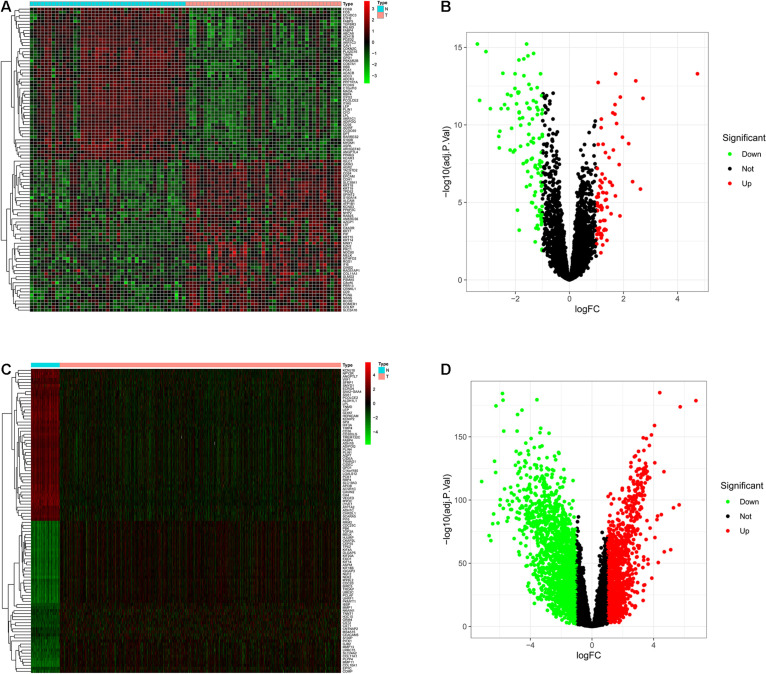
The differentially expressed genes (DEGs) were screened. Each column of the heat map represents the sample, the row represents the gene, and each grid represents the degree of gene expression in the sample. The row of the volcano graph represents log|FC|, and the column represents −log10 (adjusted *P*-value), and each point is the degree of gene expression. **(A)** Heat plot of DEGs in the GEO. **(B)** Volcano plot of DEGs in the GEO. **(C)** Heat plot of DEGs in the TCGA. **(D)** Volcano plot of DEGs in the TCGA.

DEGs analysis can be used to screen out the differential genes between cancer tissues and normal tissues. Heat plots of GSE15852 and TCGA-BC (Figures 3A,C) were drawn. In the heat plot, each cell represents the degree of gene expression, red represents up-regulation, and green represents down-regulation. We take log|FC| as the horizontal axis and −log_10_(adj. *P*-value) as the vertical axis to make volcano plots (Figures 3B,D). In the volcano plot, red and green points are the differential genes. They were screened out based on |logFC| ≥ 1.0 and (adjusted *P*) < 0.05. Finally, 739 differential genes of GEO and 1,290 differential genes of TCGA were obtained.

**FIGURE 4:**
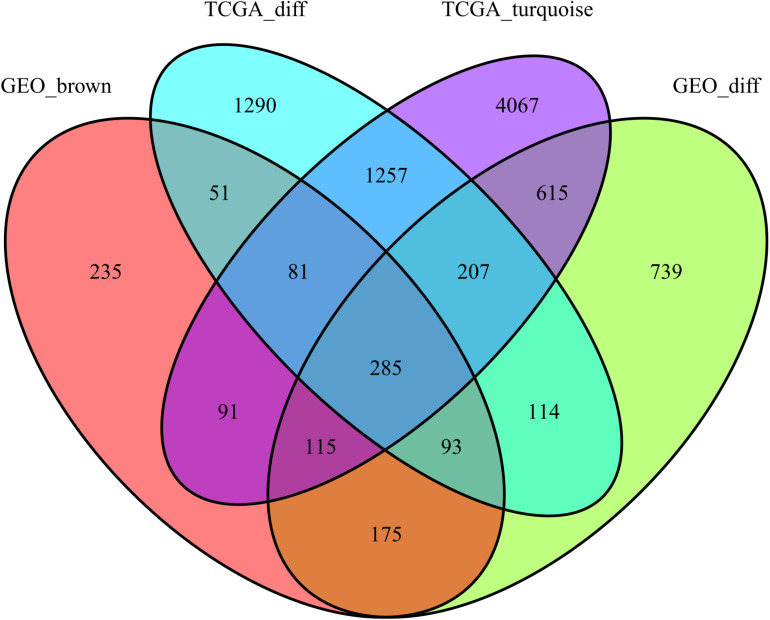
Gene Venn diagrams between the two groups of DEGs and the two groups of co-expressed genes.

**FIGURE 5:**
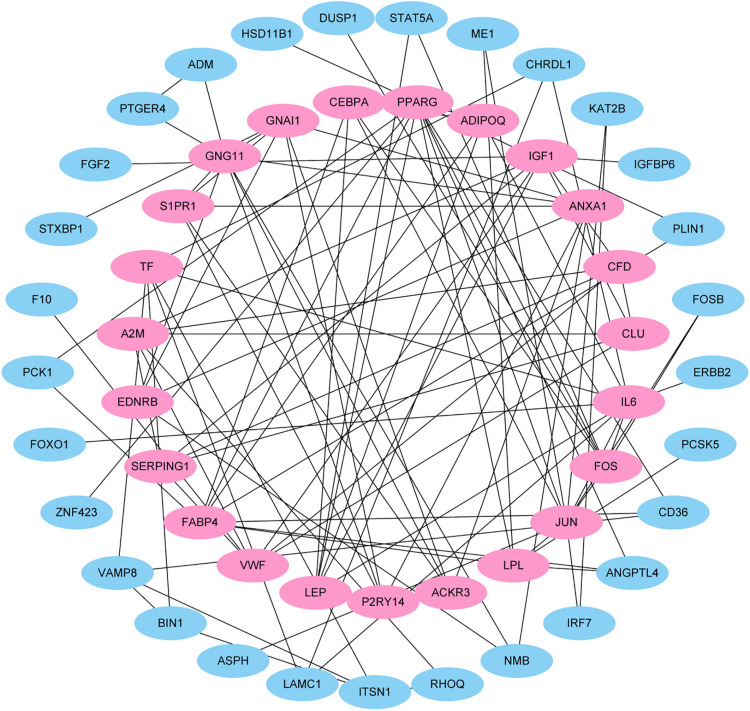
Protein–protein interaction (PPI) network. Each node in the figure represents a protein, and the edge represents the interaction between the two proteins.

This study extracted the overlapping genes of the above four groups of genes, and the R package Venn diagram was used to visualize the overlapping genes (Figure 4). We built a protein interaction network of overlapping genes (Figure 5) and the PPI network was used to extract the hub genes. The hub genes were screened from the PPI network based on the MCC. The MCC and degree of these genes were listed in Table 1. The pink nodes in Figure 5 are hub genes. Twenty-three genes were extracted including GNG11, ANXA1, GNAI1, IGF1, VWF, A2M, ACKR3, P2RY14, S1PR1, CFD, CLU, SERPING1, PPARG, CEBPA, FABP4, JUN, ADIPOQ, EDNRB, TF, IL6, FOS, LPL, and LEP. The hub genes were submitted into DAVID 6.8^3^ for KEGG pathway analysis. KEGG analysis revealed that hub genes were mainly enriched in “Pathways in cancer” (Table 2).

**TABLE 1:** Maximal clique centrality and degree of hub genes.

Node name	MCC	Degree	Node name	MCC	Degree
GNG11	134	9	PPARG	34	13
ANXA1	132	7	CEBPA	18	6
GNAI1	127	7	FABP4	18	8
IGF1	123	8	JUN	17	10
VWF	121	6	ADIPOQ	14	5
A2M	121	6	EDNRB	12	4
ACKR3	120	5	TF	12	6
P2RY14	120	5	IL6	12	9
S1PR1	120	5	FOS	12	7
CFD	120	5	LPL	11	6
CLU	120	5	LEP	10	6
SERPING1	120	5			

**TABLE 2:** Pathway enrichment analysis of hub genes.

KEGG pathway ID and term	Count	*P*-value	Genes
hsa05200: Pathways in cancer	9	7.25 × 10^–6^	CEBPA, IL6, JUN, EDNRB, PPARG, FOS, IGF1, GNG11, GNAI1
hsa05133: Pertussis	5	5.53 × 10^–5^	IL6, JUN, SERPING1, FOS, GNAI1
hsa04932: Non-alcoholic fatty liver disease (NAFLD)	5	8.22 × 10^–4^	CEBPA, IL6, JUN, LEP, ADIPOQ
hsa03320: PPAR signaling pathway	4	8.94 × 10^–4^	FABP4, ADIPOQ, LPL, PPARG
hsa04610: Complement and coagulation cascades	4	9.74 × 10^–4^	CFD, VWF, SERPING1, A2M
hsa05142: Chagas disease (American trypanosomiasis)	4	3.17 × 10^–3^	IL6, JUN, FOS, GNAI1
hsa04152: AMPK signaling pathway	4	5.09 × 10^–3^	LEP, ADIPOQ, PPARG, IGF1
hsa05202: Transcriptional misregulation in cancer	4	1.18 × 10^–2^	CEBPA, IL6, PPARG, IGF1
hsa05132: Salmonella infection	3	2.37 × 10^–2^	IL6, JUN, FOS
hsa05323: Rheumatoid arthritis	3	2.65 × 10^–2^	IL6, JUN, FOS

We take the expression of 23 genes as the input of the model, and then get the classification results. The accuracy of each model in diagnosing BC is shown in Table 3. To choose the best model, SVM and ANN were selected for comparative analysis. We set the parameters of the two models as follows.

**TABLE 3:** Accuracy results of each model.

Model	First (%)	Second (%)	Third (%)	Average (SD)
SVM	97.28	96.73	96.46	96.82% (±0.34%)
ANN	97.82	97.00	97.27	97.36% (±0.34%)
CNN (LeNet)	91.01	89.65	90.46	90.37% (±0.56%)
CNN (AlexNet)	91.82	90.46	91.55	91.27% (±0.59%)
BN	93	93	93	93% (0)
DT	95.6	95.3	94.8	95.23% (±0.33%)

The range of the initial penalty parameter C of SVM was [−5, 15], the range of the kernel function parameter g was [−9, 3], and the parameters were optimized through ten-fold cross-validation.

Artificial neural network had four layers, and the number of nodes in each layer was 23, 10, 2, and 1, respectively. The first is the input layer, the second and third are the hidden layer, and the fourth is the output layer. The optimization algorithm was L-BFGS, and the learning rate was e^–5^.

**FIGURE 6:**
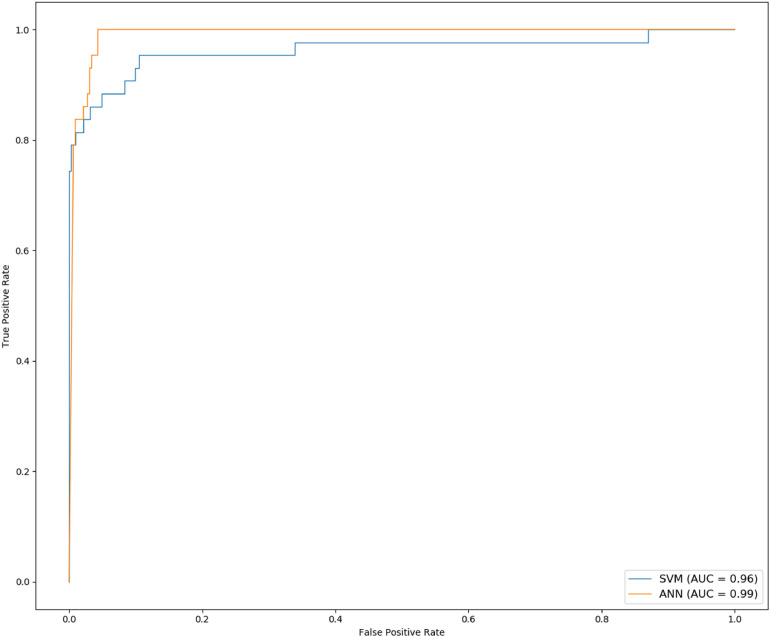
The ROC and AUC of ANN and SVM.

Ten experiments were performed for each model. The average value of F1, sensitivity and specificity was taken. The F1 of SVM is 0.8176 (±0.0477), the sensitivity is 97.69% (±0.88%), and the specificity is 83.80% (±4.64%); the F1 of ANN is 0.8535 (±0.0260), the sensitivity is 98.32% (±0.32%), and the specificity is 89.59% (±3.53%). The results are shown in Table 4. The ROC curve and AUC value of SVM and ANN are shown in Figure 6. As shown in Figure 6, the AUC of SVM is 0.96, and the AUC of ANN is 0.99.

**TABLE 4:** Model metrics.

Model	F1 (SD)	Sensitivity (SD)	Specificity (SD)
SVM	0.8176 (±0.0477)	97.69% (±0.88%)	83.80% (±4.64%)
ANN	0.8535 (±0.0260)	98.32% (±0.32%)	89.59% (±3.53%)

*F1, Sensitivity and Specificity values are the average of 10 experiments.*

F1 and AUC are indicators for evaluating classification models. Sensitivity represents the ratio of correctly predicted cancer samples, and specificity represents the ratio of correctly predicted normal samples. From the experimental results, it can be seen that F1, specificity, and AUC of ANN are higher than those of SVM. So, ANN is the best for the classification of cancer samples.

## Discussion

This work innovatively combined comprehensive biological information analysis and deep learning to classify BC samples and normal samples. In our work, we screened out 23 hub genes including SERPING1 and VWF. KEGG pathway analysis demonstrated that CEBPA, IL6, JUN, EDNRB, PPARG, FOS, IGF1, GNG11, and GNAI1 were enriched in “Pathways in cancer.” In BC samples, the −log10 (*P*-value) of H19_STAT1_SERPING1 and H19_GATA2_VWF are 3.20 and 4.06 (Li et al., 2020). It is to say that SERPING1 and VWF are related to BC. In short, it is effective to classify samples based on the expression of these genes.

In the selection of classification models, we chose SVM with better performance (Huang et al., 2018), the popular deep learning models which are ANN, CNN-lenet, and CNN-AlexNet (Min et al., 2017), and other models which are BN and DT. We used the above models to classify the samples, and found that ANN performs the best. The basic unit of the ANN is neuron. To get better performance, the weight and bias of each neuron were constantly updated during training. Classification results of ANN indicated that the average accuracy is 97.36% (±0.34%), the F1 value is 0.8535 (±0.0260), the sensitivity is 98.32% (±0.32%), the specificity is 89.59% (±3.53%), and the AUC value is 0.99.

This model can be applied to the early diagnosis of cancer. In this method, probes are firstly used to measure gene expression, and then deep learning methods are used to classify cancer samples. There is no instrument contact during the whole diagnosis process, so there is no risk of radiation compared with Mammography, CT, and PET. This method also improves the sensitivity. Specifically, the sensitivity of this method is 98.32% (±0.32%), and the sensitivities of Mammography, Ultrasound and, CT diagnosis are 67.8, 83, and 91%, respectively (Wang, 2017). The classification in this article is computer-assisted, and pathological diagnosis requires manual operation throughout the entire process, so this method is more suitable for rapid diagnosis.

In future, a large amount of single-cell sequencing data needs to be researched. Research topics involve classification and clustering tasks. The deep learning method used herein may be applied to these data (Tian et al., 2019; Qi et al., 2020). With the development of sequencing data and deep learning, we can truly develop small-scale rapid detection equipment for cancer. It provides opportunities for cancer prevention and treatment.

## Data Availability Statement

Publicly available datasets were analyzed in this study. This data can be found here: TCGA (https://portal.gdc.cancer.gov/repository) GEO (https://www.ncbi.nlm.nih.gov/geo/).

## Author Contributions

ChC, CeC, FC, NZ, ZY, and XL contributed to the conception of the study. DJ analyzed the data and wrote the manuscript. ChC and XL reviewed the manuscript. XL supervised the study. All authors contributed to the article and approved the submitted version.

## Conflict of Interest

The authors declare that the research was conducted in the absence of any commercial or financial relationships that could be construed as a potential conflict of interest.

## Footnotes

1 https://portal.gdc.cancer.gov/repository

2 https://www.ncbi.nlm.nih.gov/geo/

3 https://david.ncifcrf.gov/summary.jsp
